# Body size predicts ontogenetic nitrogen stable-isotope (*δ*^15^N) variation, but has little relationship with trophic level in ectotherm vertebrate predators

**DOI:** 10.1038/s41598-024-61969-5

**Published:** 2024-06-19

**Authors:** Francisco Villamarín, Timothy D. Jardine, Stuart E. Bunn, Adriana Malvasio, Carlos Ignacio Piña, Cristina Mariana Jacobi, Diogo Dutra Araújo, Elizângela Silva de Brito, Felipe de Moraes Carvalho, Igor David da Costa, Luciano Martins Verdade, Neliton Lara, Plínio Barbosa de Camargo, Priscila Saikoski Miorando, Thiago Costa Gonçalves Portelinha, Thiago Simon Marques, William E. Magnusson

**Affiliations:** 1 Grupo de Biogeografía y Ecología Espacial (BioGeoE2), Universidad Regional Amazónica Ikiam, Tena, Ecuador; 2 School of Environment and Sustainability, University of Saskatchewan, Saskatoon, SK Canada; 3 Australian Rivers Institute, Griffith University, Nathan, QLD Australia; 4 Laboratório de Ecologia e Zoologia (LABECZ), Curso de Engenharia Ambiental, Universidade Federal do Tocantins, Palmas, TO Brazil; 5 Centro de Investigación Científica y de Transferencia Tecnológica a la Producción (Consejo Nacional de Investigaciones Científicas y Técnicas, Provincia de Entre Ríos, Universidad Autónoma de Entre Ríos), Diamante, Argentina; 6 Institute of Biosciences, São Paulo State University (UNESP), Rio Claro, Brazil; 7 Laboratório de Ecologia de Vertebrados Terrestres (LEVERT), Universidade do Vale do Rio dos Sinos, São Leopoldo, RS Brazil; 8 Laboratório de Herpetologia, Universidade Federal de Mato Grosso, Cuiabá, Brazil; 9 Virginia Polytechnic Institute and State University, Blacksburg, VA USA; 10 Instituto do Noroeste Fluminense de Educação Superior, Universidade Federal Fluminense, Santo Antônio de Pádua, RJ Brazil; 11 Wildlife Management Consultancy, Campinas, Brazil; 12 Centro de Energia Nuclear na Agricultura, Universidade de São Paulo, Piracicaba, Brazil; 13 Universidade Federal do Oeste do Pará, Campus Oriximiná, Pará, Brazil; 14 Laboratório de Caracterização de Impactos Ambientais (LCIA), Curso de Engenharia Ambiental, Universidade Federal do Tocantins, Palmas, TO Brazil; 15 Laboratório de Ecologia Aplicada, Núcleo de Estudos Ambientais, Universidade de Sorocaba, Sorocaba, Brazil; 16 Coordenação de Biodiversidade, Instituto Nacional de Pesquisas da Amazônia, Manaus, Brazil

**Keywords:** Ecology, Stable isotope analysis

## Abstract

Large predators have disproportionate effects on their underlying food webs. Thus, appropriately assigning trophic positions has important conservation implications both for the predators themselves and for their prey. Large-bodied predators are often referred to as apex predators, implying that they are many trophic levels above primary producers. However, theoretical considerations predict both higher and lower trophic position with increasing body size. Nitrogen stable isotope values (*δ*^15^N) are increasingly replacing stomach contents or behavioral observations to assess trophic position and it is often assumed that ontogenetic dietary shifts result in higher trophic positions. Intraspecific studies based on *δ*^15^N values found a positive relationship between size and inferred trophic position. Here, we use datasets of predatory vertebrate ectotherms (crocodilians, turtles, lizards and fishes) to show that, although there are positive intraspecific relationships between size and *δ*^15^N values, relationships between stomach-content-based trophic level (TP_diet_) and size are undetectable or negative. As there is usually no single value for ^15^N trophic discrimination factor (TDF) applicable to a predator species or its prey, estimates of trophic position based on *δ*^15^N in ectotherm vertebrates with large size ranges, may be inaccurate and biased. We urge a reconsideration of the sole use of *δ*^15^N values to assess trophic position and encourage the combined use of isotopes and stomach contents to assess diet and trophic level.

## Introduction

Large predators have disproportionate effects on their underlying food webs^1^. Thus, appropriately assigning trophic positions has important conservation implications both for the predators themselves and for their prey^2^. Large predators are often referred to as top or apex predators because they are not prey for other species in their food chain. As such, many assume that the largest organisms should occupy the highest trophic positions, both within and among species. Though this assumption holds in non-filter feeders in marine pelagic ecosystems where the principal primary producers are planktonic algae^3^, many food chains in other ecosystems lack or have reduced trophic size structure^4^.

Over the past 40 years, nitrogen stable isotope values have become an increasingly-used tool to unveil consumer trophic position. The mechanism underlying this application relies on organisms preferentially retaining ^15^N and excreting ^14^N, which leads to differences in the standard-normalized ratio ^15^N:^14^N (*δ*^15^N) in the tissues of consumers and their food^5^. *δ*^15^N values of a consumer are typically enriched by 3–4‰ relative to its diet^6–8^, though the value most frequently used is 3.4‰^5^. This is commonly known as the trophic discrimination factor—TDF^9^, which explicitly assumes that isotopic differences between consumer and prey are exclusively related to trophic factors and to isotopic discrimination. Although commonly described in the literature as TDF, differences between prey and predator result from many causes, such as isotopic routing (i.e. differential degrees of transamination/deamination for differing metabolites) and preferential assimilation of diet components. The term tissue-diet spacing is more inclusive of these other processes, but differences between predators and prey are generally assumed to be primarily due to isotopic discrimination. In fact, it is not possible to evaluate TDF without examining the simultaneous effects of degrees of transamination/deamination, but we are unaware of any field studies that have taken this into account. Throughout the manuscript, we use TDF for simplicity, as have most authors, but we recognize that it may result from much more than isotopic discrimination.

Many studies based on nitrogen stable isotopes have reported positive relations between consumer body size and *δ*^15^N values and consistently concluded the existence of ontogenetic increases in trophic position (e.g.,^10–13^). To this end, the latter studies calculated trophic position assuming constant TDF values. Others, have assumed that different sources result in distinct, but constant TDF^14^. Similarly, TDF and other metabolically related factors are tissue specific, but most authors have made comparisons only between samples of the same tissue type, or have calibrated the results to take into account tissue-specific factors.

Controlled feeding experiments are starting to reveal the physiological processes that govern trophic discrimination factors, assimilation, and, especially, turnover rates. There is a rich literature on laboratory-derived TDFs for species fed controlled diets, as well as meta-analyses of those studies (e.g.^15^). Most laboratory studies, however, are conducted with short-lived, fast-growing organisms that are easily kept in captivity (e.g. rainbow trout, mice). Far rarer are studies on large-bodied species with long life spans^16^. Of those few that have studied large-bodied, long-lived species, most tend to focus on estimating isotopic turnover rates after a diet switch^17,18^, and therefore calculate TDFs at equilibrium on the new and old diets, rather than comparing TDFs among different life stages on the same diet. This limits our ability to determine if TDFs vary with size/age under controlled conditions. There has been debate about the reliability of using constant TDF to determine trophic position^19–22^. It is well recognized that many factors affect TDF, including food quality, metabolic paths, developmental stage, growth rate, body mass, temperature, and sex^14,15,23–25^. In controlled studies, TDF of captive Atlantic salmon have been related to growth rate^25^, which is ultimately dependent on body size in ectotherms^26^. The authors of Reference^27^ found increasing *δ*^15^N values with age—and size—for walleye despite a constant diet. Also, ticks fed constant diets in controlled conditions had *δ*^15^N values that consistently increased as ticks aged^28^. The authors of Reference^16^ found that TDF was strongly dependent on body size of farmed-raised *Crocodylus niloticus* fed a constant diet. These observations in controlled and semi-controlled conditions suggest that tissues could accumulate ^15^N with age, confounding trophic-position estimates in long-lived organisms. There is no reported relationship between TDFs and metabolic rate for endotherms within species (e.g.^29^), but there is a strong relationship with turnover rates across species^30,31^. However, there are few studies of the physiological processes that govern trophic discrimination factors for large ectotherms.

Despite the accumulating evidence that TDF is not constant in a variety of organisms^32^, and that current Bayesian isotope models are customizable to consider some uncertainties and random variation and thus can handle varying TDF for various consumers^33,34^, most estimates of trophic level based on *δ*^15^N values have assumed that TDF remains constant across the lifespan of an organism (but see^35^).

More recent field studies have suggested that size (age) or growth rate—and all the physiological processes that might change with age—are more important than the trophic level of prey in determining *δ*^15^N values in tissues of Amazonian caimans and arapaima fish^36,37^. Those findings raise important questions about the stability of an organism’s TDF over time and point to possible changes in *δ*^15^N values due to size-related metabolic changes. As suggested by the autors of Reference^38^ and Reference^25^, high rates of catabolism (tissue-exchange) promote transamination and preferential loss of ^14^N. Therefore, TDF is expected to vary negatively with growth rate and positively with tissue catabolism in ectotherms^25^. Thus, one would predict a systematic positive co-variation between body size and *δ*^15^N values for an organism fed a constant (nutrient replete) diet through ontogeny (see^35^). If metabolic-related ontogenetic variation in TDF is more important than differences in trophic level in the field and laboratory, estimates of trophic position based on an assumed constant value of TDF between a species and its food sources may be misleading. However, to date, the identification of potential biases in field studies was based on a handful of large aquatic predators, and it is unknown to what extent such biases occur in other ectotherm vertebrate consumers, including both aquatic and terrestrial species.

In this study, we use datasets on tropical and sub-tropical ectotherm vertebrate predators and omnivores (crocodilians, turtles, lizards and fishes) to determine how body size impacts their inferred trophic position. We determined the extent that differences in *δ*^15^N values and stomach-content-derived trophic position (TP_diet_) reflect shifts in the size of consumers, which we assume is a surrogate for metabolic processes, and evaluated if and how differences in *δ*^15^N values reflect shifts in TP_diet_.

## Results

**Figure 1:**
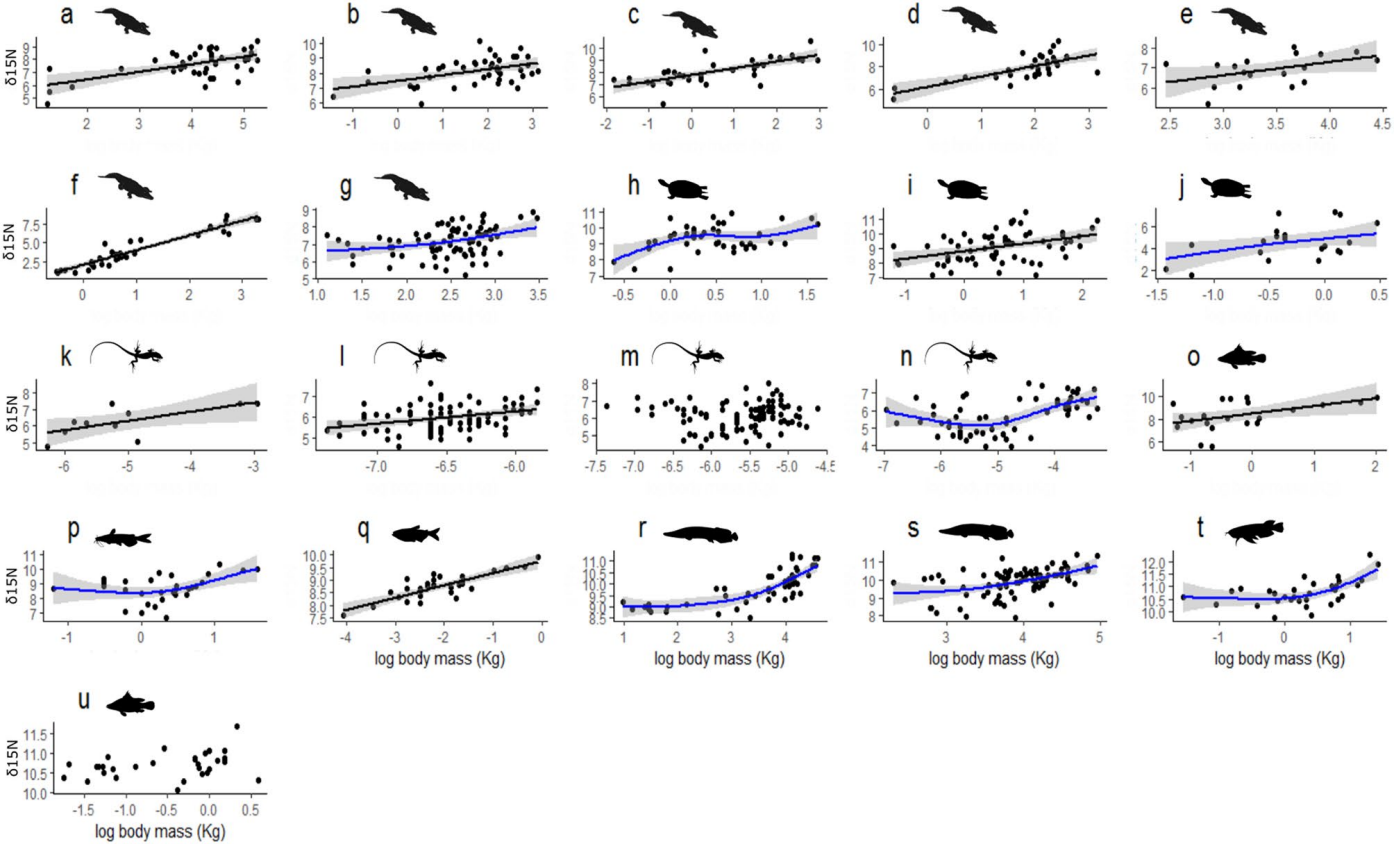
Increases in *δ*^15^N values as a function of log-transformed body mass for species with stable isotope data available. Black and blue trend lines represent GLM and GAM models, respectively, with 0.95 confidence intervals (shaded area). Due to orders-of-magnitude differences in body size among the studied organisms, axes ranges are not standardized across panels. **Crocodilians:** (**a**) *Crocodylus porosus,* (**b**) *Paleosuchus trigonatus*^36^*,* (**c**) *P. palpebrosus*^36^*,* (**d**) *Caiman crocodilus*^36^*,* (**e**) *C. latirostris 1,* (**f**) *C. latirostris 2*^64,65^, (**g**) *C. latirostris 3;* **Turtles:** (**h**) *Podocnemis unifilis 1,* (**i**) *P. unifilis 2,* (**j**) *Mesoclemmys vanderhaegei;* **Lizards:** (**k**) *Ameiva ameiva,* (**l**) *Anolis auratus,* (**m**) *Cnemidophorus lemniscatus,* (**n**) *Kentropyx striata;* **Fishes:** (**o**) *Lates calcarifer,* (**p**) *Neoarius leptaspis,* (**q**) *Hoplias malabaricus,* (**r**) *Arapaima 1*^11^*,* (**s**) *Arapaima 2*^37^*,* (**t**) *Osteoglossum bicirrhosum,* (**u**) *Cichla* sp.

*δ*^15^N values co-varied with body size for most consumers. Most species/populations (19 of 21) with *δ*^15^N values available showed positive relationships with strong to moderate statistical support between log body mass and *δ*^15^N values. Only two species (*Cnemidophorus lemniscatus*, rainbow whiptail lizard and *Cichla* sp., peacock bass) showed no evidence for such a relationship (Fig. 1; Supplementary Table S1). When all species were pooled in the same analysis by removing the effect of the species identity, a GLM showed an increase of standardized *δ*^15^N values as a function of standardized body mass (LogLik = − 1136.8, Deviance = 626.8, df.null = 930, df.residual = 929, pseudo r^2^ = 0.25, p < 0.001; Supplementary Fig. S1).

**Figure 2:**
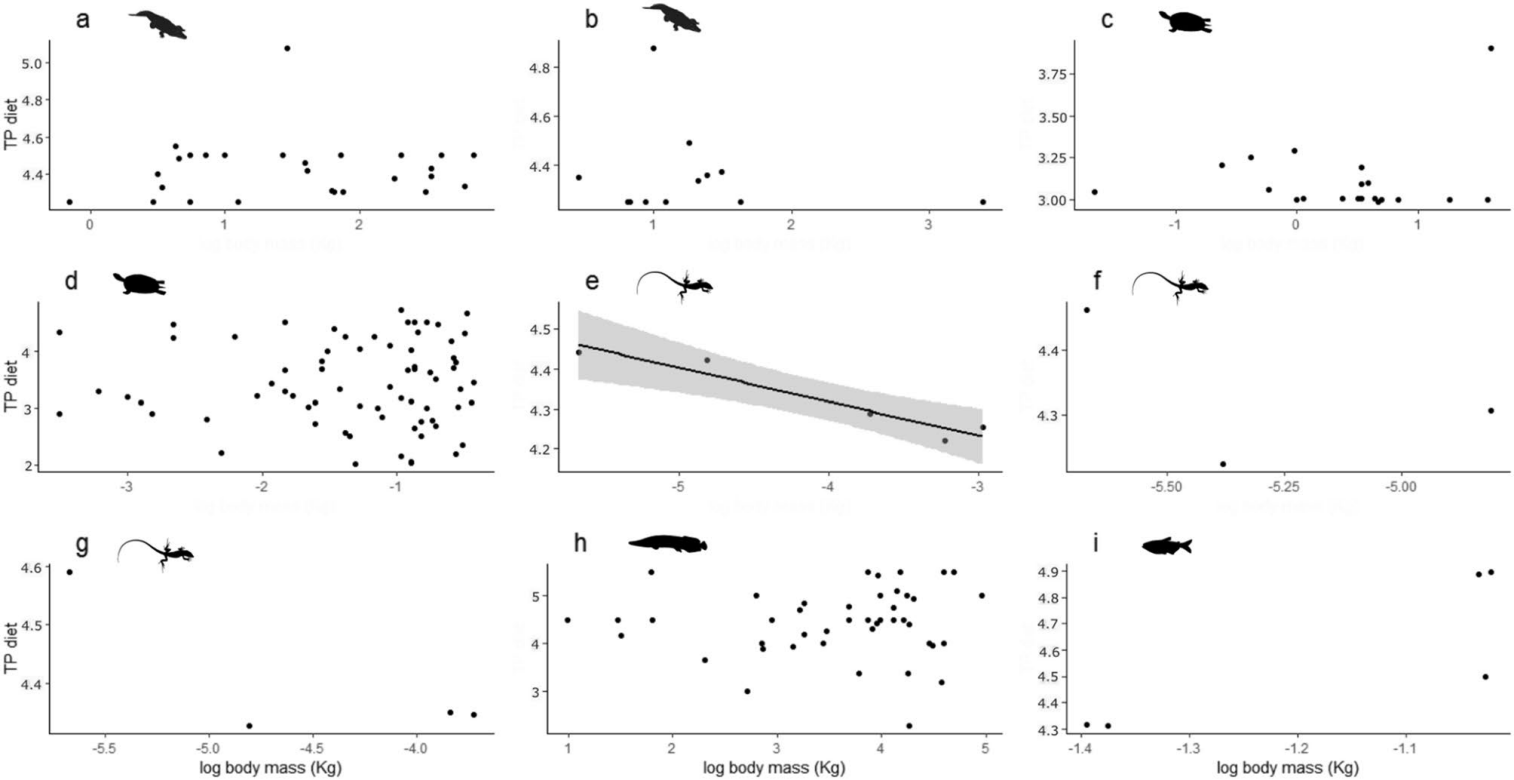
Relationships between stomach-content-derived trophic position (TP_diet_) and log-transformed body mass for species with dietary data available. Data are for individuals except for the lizards in which points represent mean values of body mass and TP_diet_ for size classes. Black trend line represents a GLM model with 0.95 confidence interval (shaded area). Due to orders-of-magnitude differences in body size among the studied organisms, axes ranges are not standardized across panels. **Crocodilians:** (**a**) *Caiman crocodilus,* (**b**) *Melonosuchus niger;* **Turtles:** (**c**) *Podocnemis unifilis,* (**d**) *Mesoclemmys vanderhaegei*^72^*;* **Lizards:** (**e**) *Ameiva ameiva*^59–61,73^*,* (**f**) *Cnemidophorus lemniscatus*^59–61,73^*,* (**g**) *Kentropyx striata*^59–61,73^ **Fishes:** (**h**) *Arapaima 1*^11^*,* (**i**)* Hoplias malabaricus.*

The species for which diet data were available (N = 9) showed no relationship between log body mass and TP_diet_ in 8 cases. The only statistically significant, but negative, relationship was found for the lizard *Ameiva ameiva* (Fig. 2; Supplementary Table S2).

**Figure 3:**
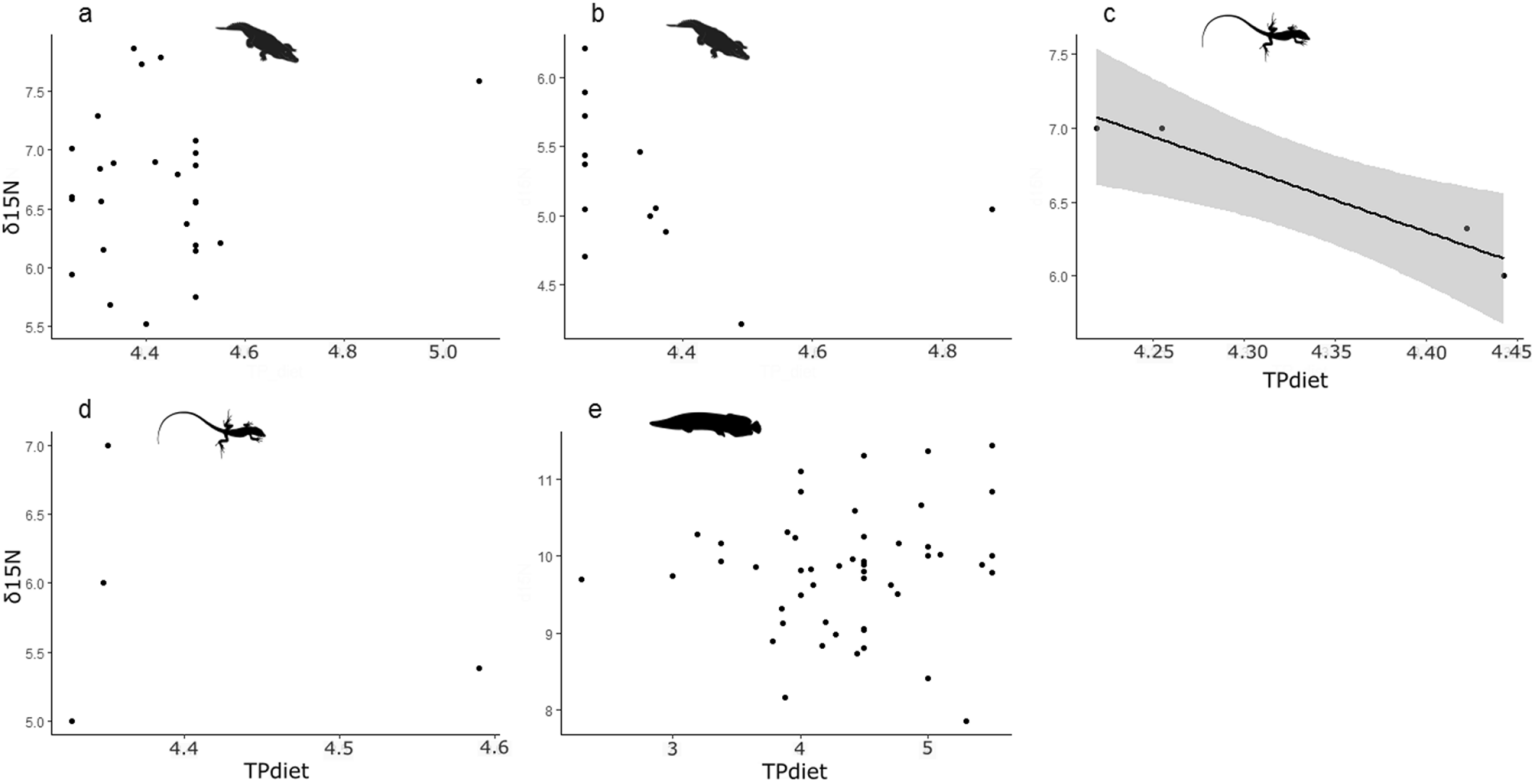
Relationships between stomach-content-derived trophic position (TP_diet_) and *δ*^15^N values for species with both stable isotopes and dietary data available. Black trend line represents a GLM model with 0.95 confidence intervals (shaded area). Due to orders-of-magnitude differences in body size among the studied organisms, axes ranges are not standardized across panels. Data are for individuals except for the lizards in which points represent mean values of body mass and *δ*^15^N values for size classes. **Crocodilians**: (**a**) *Caiman crocodilus,* (**b**) *Melanosuchus niger,* **Lizards:** (**c**) *Ameiva ameiva,* (**d**) *Kentropix striata*^59–61,73^ **Fishes:** (**e**) *Arapaima*^11^*.*

**Figure 4:**
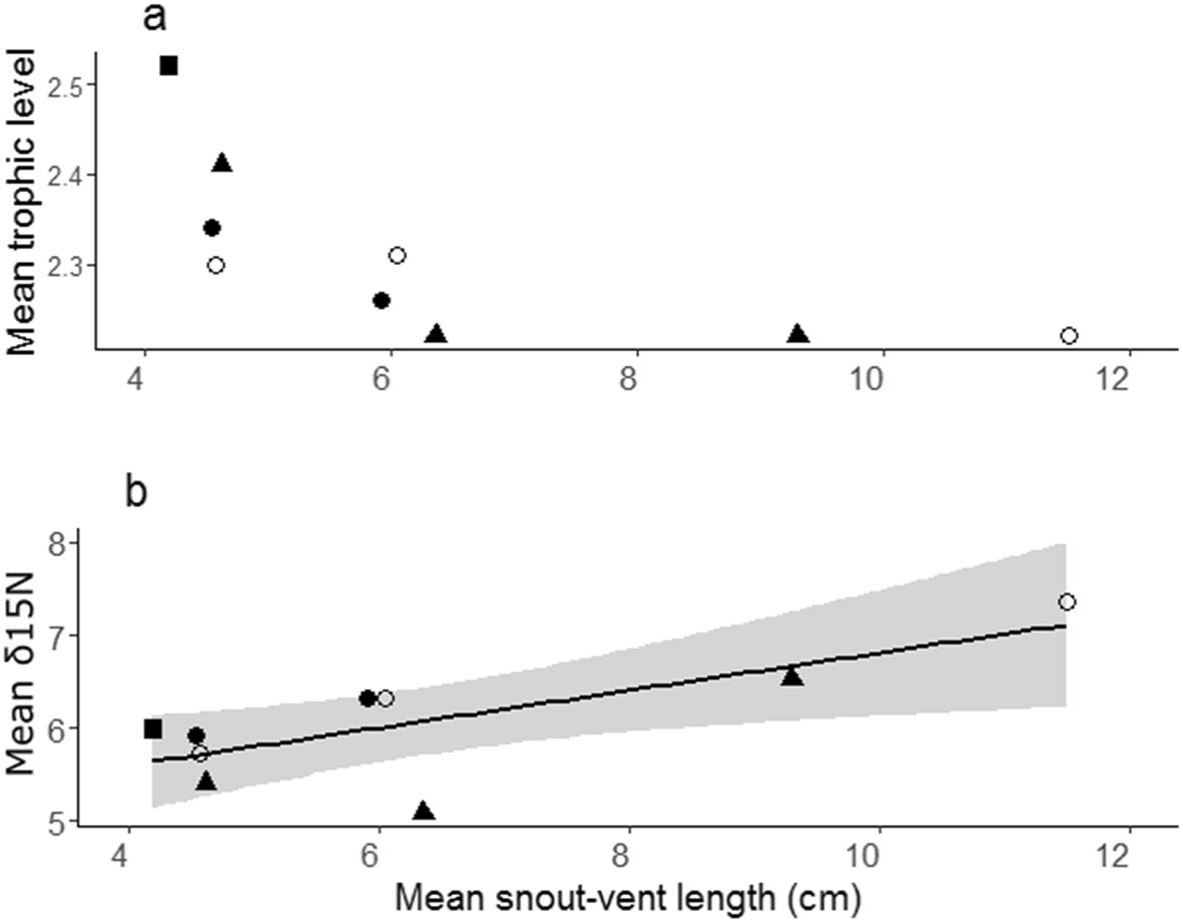
Relationship between mean snout-vent length within size categories of lizards collected in 1984–1985 and (**a**) mean trophic level by mass of their prey; and, (**b**) mean *δ*^15^N values of muscle tissue of lizards in the same size category. ○ = *Ameiva ameiva*; ■ = *Anolis auratus*; ● = *Cnemidophorus lemniscatus*; ▲ = *Kentropix striata*.

Similarly, differences in *δ*^15^N values did not reflect shifts in stomach-content-derived trophic position. Within the species for which both *δ*^15^N values and stomach-content data were available (N = 5) there was no significant relationships between TP_diet_ and* δ*^15^N values, except for the lizard *A. ameiva*, which showed a very strong negative relationship (Fig. 3; Supplementary table S3). Though the latter was based on only four data points, these were means of many individuals within size classes and the general pattern is similar to that when data for this species is included with other lizards from the same area (Fig. 4).

For the data on the lizard assemblage from 1984 to 1985, the variables that represent species differences in TDF (species), geographic differences in baseline (plot) and differences in baseline due to foraging strata (*δ*^13^C) explained much of the differences among individuals in *δ*^15^N values (R^2^ = 0.45). All variables contributed significantly to the analysis (species: F_3,204_ = 12.28, P < 0.001; plot: F_39,204_ = 3.34, P < 0.001; *δ*^13^C: F_1,204_ = 37.7, P < 0.001). Residuals from this analysis (Res *δ*^15^N) which presumably represent variation in *δ*^15^N values independent of baselines or species-specific TDF, were weakly, but positively, associated with snout-vent length (SVL—cm) of lizards (Res *δ*^15^N = − 0.29 + 0.055*SVL, r^2^ = 0.026, F_1,246_ = 6.54, P = 0.011).

For data from 1984 to 1985 grouped by size classes used in the diet analyses, mean trophic level of prey decreased monotonically with SVL (Fig. 4a), but mean *δ*^15^N values increased with SVL (Fig. 4b). Analysis of covariance indicated that values of *δ*^15^N values were related to species (F_3,2_ = 62.9, P = 0.016), mean trophic level of stomach contents (F_1,2_ = 43.6, P = 0.022) and the natural logarithm of mean SVL (F_1,2_ = 109.8, P = 0.009), but there was little evidence for an effect of *δ*^13^C for the aggregated data (F_1,2_ = 6.1, P = 0.130). The complete model explained most of the variance in the data (r^2^ = 0.996). After consideration of the other variables, trophic level of stomach contents contributed an extra 0.086 to the R^2^, whereas mean SVL had an independent contribution (0.215) that was 2.5 times greater. As all lizards were collected in the same area and there was no evidence for a relationship with *δ*^13^C, variation in baselines is unlikely to affect these results.

## Discussion

Examination of stomach contents to determine the trophic level of food items is time consuming and gives only information on what the animal had eaten in the recent past. In addition, the mechanical triage of food contents tends to be biased toward indigestible items, such as bones, feathers, and hair, which can result in an uneven detection rate among distinct food items (e.g.,^39^). Inferring the trophic level of each category of food is subjective and introduces a further source of uncertainty. Therefore, researchers have sought alternative methods to determine the trophic levels of organisms in food webs. However, it is important that these methods are at least as good at estimating trophic level as examination of stomach contents and not just more sophisticated in the original sense of the word, which was “unnecessarily complicated”.

The use of *δ*^15^N values has become the method of choice to determine trophic level in most food-web studies, but it is important to stress that use of *δ*^15^N values was justified on its relationship with stomach-contents analyses (e.g.,^40^). Where discrepancies between stomach content and isotope data exist, a strong relationship between size and *δ*^15^N values is commonly used as evidence supporting isotope-based trophic level as more accurate than stomach content data. However, if this is a mechanistic effect of growth/metabolism then that observation is misleading. Thus, if both methods produce divergent results, the assumption that *δ*^15^N values are always an accurate index of trophic position needs to be re-evaluated. In some cases, information on stable isotopes has indicated probable biases in estimates of trophic level based on stomach contents, finding agreement between both methods for some trophic guilds of fish but not for others^41^. Intraspecific studies have generally either ignored what was known about the trophic level of prey (e.g.,^13^), or assumed that stomach contents do not reflect what is incorporated in the predator because of differential digestibility and assimilation (e.g.,^14^). Very few have concluded that the gut-content analyses are essential complements to the isotope estimates of trophic position (e.g.,^42^). That is, *δ*^15^N values have become the de facto standard for determining the trophic level of organisms in food-web studies^43^ and direct observations of diet, when available, are given less importance unless being used as prior information in Bayesian models with stable isotopes. However, we found that stomach-content and nitrogen stable-isotope analyses can lead to different conclusions unless the effect of body size is taken into account.

Most recent studies that have evaluated the trophic level of organisms have acknowledged the sources of bias listed in the introduction (i.e. non-constant TDF^14,15,23–25^), but have continued to use *δ*^15^N data to estimate trophic level, presumably because they assume that variation due to these factors in the field is trivial in relation to the effects of the differences between organisms and their foods, or because they believe that the biases will be subsumed in the variance components of Bayesian analyses. However, studies of intraspecific variation in crocodilians^16,36^ and a large fish^37^ indicate that physiological factors associated with size or growth rate, which are presumably related to metabolism, are more important than diet in determining the values of *δ*^15^N in body tissues. The likely mechanism (transamination and preferential loss of ^14^N) by which catabolism affects nitrogen fractionation, predicts a systematic relationship between body size and tissue *δ*^15^N values that is independent of trophic level^25,38^.

The present study reinforces this for a wider range of organisms. We report on relationships for ectothermic predators in which age and size are strongly linked, but the processes are general and probably apply to all groups. Endotherms may show less within-individual variation in metabolism associated with size and age, but many or most endotherms at high trophic levels feed on ectotherms, so the results of this study will be relevant for estimates of their trophic level using stable isotopes.

Independent data collected in different sites and species indicate that intraspecific differences in size (and physiological factors associated with it) are more important than diet in determining *δ*^15^N values in vertebrate ectotherm predators. These findings hold for both intraspecific and interspecific comparisons.

At the interspecific level, using extensive global data sets, the authors of Reference^44^ and Reference^3^ found contradictory outcomes when relating body mass to diet or *δ*^15^N-derived trophic-position estimates, respectively. Dietary data suggest that body size is not an effective predictor of trophic position in fish, and other morphological characters seem more important^44–46^. In contrast, body size and *δ*^15^N-based trophic level show a positive relationship across consumers in freshwater ecosystems^3^. Thus, similar to our results at the intraspecific level, body size seems also to be more important than diet in driving shifts in *δ*^15^N values interspecifically, at global scales.

If the effects of size only introduced a random element into estimates of trophic level, they would be less important, but they often bias the analysis. The present and previous studies (i.e.,^36,37^) indicate that the trophic level of prey eaten by predators reduces or remains constant as the animal grows, when based on stomach-content analyses. However, data from *δ*^15^N values indicate that trophic level increases with size, both within and among species, even when shifts in *δ*^15^N values over some very large size ranges are very small (~ 0.5 trophic levels), considered ecologically relevant elsewhere (e.g.,^47,48^). That is, isotope data perpetuate the potentially false assumption that the largest predators are most likely to occupy the highest trophic positions, and the physiological mechanisms operating on the nitrogen isotopic composition of ectotherms is generally underappreciated in the literature. Based on *δ*^15^N values, the authors of Reference^49^ concluded that the extinct megatooth shark, *Otodus megalodon*, “was at a high trophic level that is not represented in modern ocean food webs”, but our analyses indicate that its higher *δ*^15^N values were more likely due to its larger size than modern sharks. Even switches to herbivory later in life can be masked by the effect of size on *δ*^15^N values, as illustrated by Reference^42^ for grunters in northeast Australia.

The effect of body size is not trivial. It is much larger than the effect of the diet trophic level (e.g.,^28^); perhaps larger than our analyses indicate, because the positive effect of size has to overcome the negative effects of changes in trophic level on *δ*^15^N values. Size, or the metabolic processes for which it is a surrogate, must be taken into account when evaluating or estimating TDF for trophic-level studies for long-lived predators with large size ranges. Studies using Bayesian models to estimate food sources currently also assume constant TDF (with associated error) and are subject to the same biases. Producing such estimates will require creative, long-term laboratory studies where predators are fed a constant diet for long periods. We are aware of only one study where farmed crocodiles fed the same lifelong diet were used to evaluate body size-driven shifts in TDF^16^. Although this was not a controlled feeding experiment, diet was assumed to remain constant throughout the crocodiles’ lifespans and the TDF was strongly dependent on body size. A further complication is that ^15^N may remain in tissues long after the animal has changed diet, so diet-change experiments will be necessary to estimate the refractory period in different tissues for long-lived organisms. Even where TDF obtained in controlled experiments are available, these may not be directly comparable to TDF in the wild if tissue-diet isotopic spacing depends more on growth rates than absolute size^25^. Future laboratory studies should include variation in feeding regimes to distinguish the effects of growth rate and size. Furthermore, amino-acid compound-specific isotopic analyses are proving to be useful tools to disentangle the influences of *δ*^15^N_baseline_ values and trophic fractionation on consumer nitrogen isotope values^50–52^. However, while they may resolve the baseline effect, trophic level estimation from *δ*^15^N-AA analyses still requires assumptions of fixed tissue-diet spacing between source and trophic amino acids, and it has been shown that TDF can change based on diet quality^53^. Until we have age-specific data, more-realistic estimates of trophic level using *δ*^15^N values will require more-complex analyses.The authors of Reference^14^ have taken a step in that direction by using different TDF for plant and animal foods. However, differences among species, or size classes within species, may be greater than the difference between plants and animals. Estimation of trophic levels based on *δ*^15^N values might require modelling tissue-diet spacing at the individual level, based on age/size-growth curves (e.g. proposed in the IsoDYN framework^35^). No study of trophic level using stable isotopes has attempted to fulfill this requirement. Until we have such data, estimates of trophic level based on direct observations of foods ingested, whether by stomach-content analyses or analyses of DNA^54^ are likely to provide equally valuable information as *δ*^15^N values, especially for long-lived predators. Whole-food-web studies that include both stable isotopes and stomach contents are needed to understand where and when these two methods agree and where they diverge.

We must be careful not to underestimate the value of observational data on the comprehension of trophic studies as Natural History is still fundamental to our understanding of the actual world. Efforts to protect or recover endangered predators depend on accurate information about their trophic level so that actions can be directed to food sources on which they are most dependent^55^. Attempts to bolster all trophic levels may be much less effective than recovering populations of the herbivores at low trophic levels on which most large predators appear to depend.

## Methods

### Datasets

We analyzed original and, in some cases, re-analyzed published datasets of both aquatic and terrestrial ectotherm consumers, including crocodilians (five species, seven populations), turtles (three species, four populations), lizards (four species, four populations) and fishes (six species, eight populations). We used body mass as a measure of body size for most organisms, except lizards and *Crocodylus porosus* for which we converted snout-vent length to mass using published allometric relationships^56,57^.

The data on terrestrial lizards were originally collected for studies of behavior^58^, reproduction^59^, thermoregulation^60^, diet^61^ and energy flow^62^, and we did not closely evaluate the *δ*^15^N values at the time because we assumed that it would closely track the data on diet. It was only after recent studies questioned that assumption that we realized that the data set provides a unique opportunity to investigate intra- and interspecific variation in *δ*^15^N values in an assemblage of small terrestrial predators while controlling for baseline *δ*^15^N values and possible differences in primary producers at the base of food chains. This more detailed analysis of a lizard assemblage sharing a similar habitat was based on lizards collected in a patch of savanna near the village of Alter do Chão (Brazilian Amazonia).

Lizard snout-vent length was measured with Vernier calipers. Samples for isotope analyses were collected between July 1997 and May 1998 in 38 plots distributed throughout the savannas of Alter do Chão^62^. Each plot covered approximately four hectares.

For the broader dataset, according to their availability, we used data on organisms for which only stable-isotope data were available (SIA data), organisms with stomach-content information available (diet data), and organisms for which both SIA and diet data were available for the same individual.

The studies sampled organisms at different times and sites, and used different capture methods, stomach-contents collection procedures, and tissue types for stable-isotope analysis (Supplementary Table S4). Therefore, the datasets used reflect the diversity of methods that have been used to infer trophic position from *δ*^15^N values in previous studies. However, the stomach-contents we used for each species were collected over the same season and from the same habitat. All applicable institutional and/or national guidelines for the care and use of animals were followed.

### Nitrogen stable-isotope analysis

Stable-nitrogen-isotope analyses were undertaken using mass spectrometry, which measures the ratio of heavy and light isotopes (^15^N/^14^N) of the studied samples in relation to atmospheric nitrogen. Isotopic ratios (*δ*) are expressed in parts per mil (‰), defined as *δ* (‰) = ((R_sample_/R_standard_) − 1), where R_sample_ and R_standard_ are the isotopic ratios of the sample and the standard, respectively.

Published *δ*^15^N data^11,36,37,63,64^, published databases^65^ and original data on crocodilians, turtles, lizards, and fishes were included in the analyses. In these studies, the *δ*^15^N data were collected specifically to evaluate trophic position, but in most cases baseline *δ*^15^N data were not collected (except for lizards) and/or possible differences in basal sources were not controlled, which can bias interpretation of *δ*^15^N data^66^, especially since organisms can switch from low to high baseline *δ*^15^N values or high to low *δ*^15^N baseline values throughout their lifespan^67,68^.

For the lizard-assemblage data, isotopic estimates of trophic level were based on the difference between *δ*^15^N values of the organisms and baseline values of primary producers or primary consumers. Baseline values differ among localities and the strata from which the food is taken. We do not know the baseline values for each plot, but we included plot as a categorical variable to account for geographic differences in baseline values. In aquatic organisms, baseline values may differ spatially, depending on whether the organism forages in benthic or pelagic strata or between terrestrial and algal sources, and values of *δ*^13^C are generally used to indicate the strata from which the food was obtained^66^. In savanna, *δ*^13^C can be used to infer whether organisms are part of food chains based on grasses and sedges (often C4 plants) or food chains based on dicotyledonous plants (C3)^62^. In this study, for the data from 1997 to 1998, we included *δ*^13^C values of tissue as an indicator of foraging stratum. Our analyses therefore ask how much species, size, and diet affect *δ*^15^N values independent of differences in baselines. All lizards were collected in the same area in 1982 and 1984–85, so there were no geographical differences in baseline, but we have no data on *δ*^13^C for the animals collected in 1982.

Data on diets of the lizards collected in the 1984–1985 studies were based on the mass of different components of stomach contents for four size categories of lizards^61^. Category 1 included all four species, category 2 included *Anolis auratus*, *Kentropyx striata* and *Ameiva ameiva*, category 3 included only *K. striata* and category 4 included only *A. ameiva*. For comparisons with the 1982 study, based only on adults, category 1 represents *A. auratus*, category 2 *C. lemniscatus*, category 3 K*. striata* and category 4 *A. ameiva*. Analyzing by size category eliminates one of the main problems in relating stomach contents to tissue *δ*^15^N values. Stomach contents of an individual are a poor indication of the general diet because insectivores generally only have one or a few prey species in the stomach, and these have not yet been assimilated. In contrast, *δ*^15^N values represent what was eaten during the preceding weeks or months. By using categories that contain multiple individuals collected throughout the year, the diet information indicates what the species has been eating throughout the full seasonal cycle and reduces the between-sample variance. Therefore, both the *δ*^15^N values and the stomach contents represent mean long-term diet.

### Estimates of stomach-content-derived trophic position (TP_diet_)

Overall, food items in the stomachs of all organisms were separated, weighed (wet mass) and identified to the lowest possible taxonomic level using taxonomic keys. Based on the assumed trophic level and the proportions of each prey type present in the stomachs, we estimated trophic position of predators using the following equation from Reference^69^:

$$TPdiet = \left[ {\sum\nolimits_{j = 1}^{n} {Pj*TLj} } \right] + 1$$

Here, the trophic position is the sum of the proportion of each prey-type category (j) in the predator diet (P_j_) multiplied by the trophic level of each prey-type category (TL_j_) and (n) is the total number of different prey types in the stomach.

Trophic level of prey was assigned according to Reference^37^ as follows: plants = 1, herbivores = 2, detritivores that consume mostly organic matter from primary producers = 2, detritivores that consume mostly from trophic levels higher than primary producers = 2.5, omnivores = 2.5, carnivores = 3 and carnivores that sometimes can eat other predators = 3.5. For prey that we were unable to identify at the species level, we estimated values according to the species most probable for the region.

### Statistical analyses

All statistical analyses and graphics were done with R software^70^. Silhouettes in figures were downloaded from http://www.phylopics.org^71^.

We used generalized linear models (GLMs, using MuMIn package) or generalized additive models (GAMs, with mgcv package) (the latter when linearity assumptions were not met) to evaluate how *δ*^15^N values and stomach-content-derived trophic position (TP_diet_) vary as a function of log-transformed body mass in each predator species. To evaluate how *δ*^15^N values vary as a function of body mass in all studied organisms pooled in the same analysis, we removed the effect of species identity by estimating standardized body mass and standardized *δ*^15^N values. That is, we subtracted the mean body mass or *δ*^15^N values of all individuals from individual body mass or *δ*^15^N values (Standardized body mass or *δ*^15^N = individual body mass or *δ*^15^N – mean body mass or *δ*^15^N). With this standardized data, we ran a GLM. We did not use z-standardization to retain the original magnitudes.

For animals in which both SIA and stomach-content data were available, we evaluated the influence of stomach-content-derived trophic position (TP_diet_) on *δ*^15^N values.

### Ethics approval

All applicable institutional and/or national guidelines for the care and use of animals were followed. Ethics Committee of the Instituto Nacional de Pesquisas da Amazonia (INPA) No. 024/2013 and 040/2018. Griffith University’s Animal Ethics Committee. Permit number: ENV/08/11/AEC. “NABH-Northern Australia Biodiversity Hub”.

### Supplementary Information

Supplementary Figure S1.

Supplementary Table S1.

Supplementary Table S2.

Supplementary Table S3.

Supplementary Table S4.

## Data Availability

Data sets utilized for this research are summarized in Supplementary Table S4. Of these, unpublished datasets were deposited in the Biodiversity Research Program repository (https://ppbiodata.inpa.gov.br/metacatui/#view/PPBioAmOc.672.2).
