# Supplementary material for: Body size predicts ontogenetic nitrogen stable-isotope (δ15N) variation, but has little relationship with trophic level in ectotherm vertebrate predators
Source: Sci Rep. 2024 Jun 19;14:14102. doi: 10.1038/s41598-024-61969-5 (PMC11189434; doi:10.1038/s41598-024-61969-5)
Supplement: Supplementary file 1 — Supplementary Figure S1. [file 41598_2024_61969_MOESM1_ESM.pdf]

Supplementary Figure S1

Body size predicts ontogenetic nitrogen stable-isotope ($\delta^{15}N$) variation, but has little relationship with trophic level in ectotherm vertebrate predators

Scientific Reports

Francisco Villamarín[1,2]; Timothy D. Jardine; Stuart E. Bunn; Adriana Malvasio, Carlos Ignacio Piña; Cristina Mariana Jacobi; Diogo Araújo; Elizângela Silva de Brito, Felipe de Moraes Carvalho; Igor David da Costa; Luciano Martins Verdade; Neliton Lara; Plínio Barbosa de Camargo; Priscila Saikoski Miorando; Thiago Costa Gonçalves Portelinha; Thiago Simon Marques and William E. Magnusson

[1]Universidad Regional Amazónica Ikiam. Grupo de Biogeografía y Ecología Espacial (BioGeoE[2]), Tena, Ecuador

[2]fco.villamarin@gmail.com

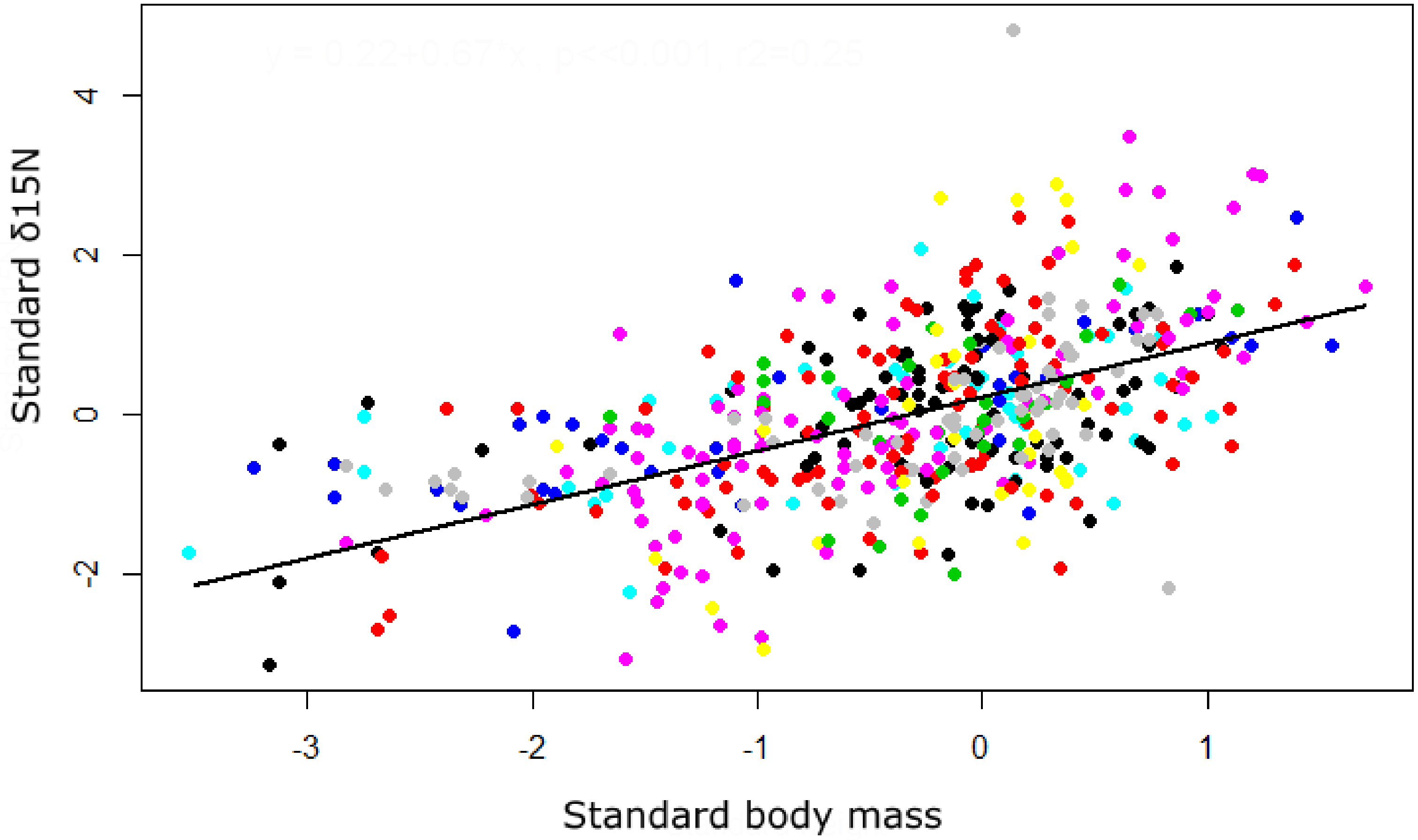

Supplementary Figure S1. Relationship of standardized δ^{15}N values as a function of standardized body mass of all species pooled in the same analysis. Colors represent each species. GLM: LogLik = -1136.8, Deviance = 626.8, df.null = 130 930, df.residual = 929, pseudo r_2 = 0.25, p <0.001.
