# Supplementary material for: Body size predicts ontogenetic nitrogen stable-isotope (δ15N) variation, but has little relationship with trophic level in ectotherm vertebrate predators
Source: Sci Rep. 2024 Jun 19;14:14102. doi: 10.1038/s41598-024-61969-5 (PMC11189434; doi:10.1038/s41598-024-61969-5)
Supplement: Supplementary file 2 — Supplementary Table S1. [file 41598_2024_61969_MOESM2_ESM.pdf]

Supplementary Table S1

Body size predicts ontogenetic nitrogen stable-isotope (δ^{15}N) variation, but has little relationship with trophic level in ectotherm vertebrate predators

Scientific Reports

Francisco Villamarín[1,2]; Timothy D. Jardine; Stuart E. Bunn; Adriana Malvasio, Carlos Ignacio Piña; Cristina Mariana Jacobi; Diogo Araújo; Elizângela Silva de Brito, Felipe de Moraes Carvalho; Igor David da Costa; Luciano Martins Verdade; Neliton Lara; Plínio Barbosa de Camargo; Priscila Saikoski Miorando; Thiago Costa Gonçalves Portelinha; Thiago Simon Marques and William E. Magnusson

[1]Universidad Regional Amazónica Ikiam. Grupo de Biogeografía y Ecología Espacial (BioGeoE[2]), Tena, Ecuador

[2]fco.villamarin@gmail.com

Supplementary Table S1. Summary statistics of GLM or GAM models evaluating how $\delta^{15}N$ varies as a function of log-transformed body mass in each predator species.

Panel in figure 1	Group	Species	Model	df	logLik	AIC	BIC	deviance	df.residual	edf	Pseudo r^2	p-value
a	Crocodilian	*Crocodylus porosus*	GLM	39.0	-51.4	108.8	113.9	30.6	38.0		0.33	<0.001
b	Crocodilian	*Paleosuchus trigonatus*	GLM	42.0	-49.9	105.8	111.1	25.7	41.0		0.22	0.002
c	Crocodilian	*Paleosuchus palpebrosus*	GLM	35.0	-38.3	82.6	87.4	17.7	34.0		0.51	<0.001
d	Crocodilian	*Caiman crocodilus*	GLM	30.0	-36.6	79.1	83.4	19.2	29.0		0.48	<0.001
e	Crocodilian	*Caiman latirostris 1*	GLM	17.0	-18.1	42.3	44.9	7.9	16.0		0.20	0.063
f	Crocodilian	*Caiman latirostris 2*	GLM	30.0	-34.2	74.3	78.6	16.4	29.0		0.90	<0.001
g	Crocodilian	*Caiman latirostris 3*	GAM	2.9	-88.2	185.5	195.9	45.4	73.1	1.92		0.001
h	Turtle	*Podocnemis unifilis 1*	GAM	4.0	-42.5	96.5	106.4	19.0	37.0	3.00		0.006
i	Turtle	*Podocnemis unifilis 2*	GLM	62.0	-84.7	175.3	181.8	54.2	61.0		0.17	0.001
j	Turtle	*Mesoclemmys vanderhaegei*	GAM	1.8	-36.7	79.3	82.4	40.5	19.2	0.82		0.030
k	Lizard	*Ameiva ameiva*	GLM	9.0	-10.1	26.3	27.2	4.5	8.0		0.44	0.036
l	Lizard	*Anolis auratus*	GLM	82.0	-73.0	151.9	159.2	28.2	81.0		0.11	0.002
m	Lizard	*Cnemidophorus lemniscatus*	GLM	94.0	-107.5	221.1	228.8	53.5	93.0		0.00	0.561
n	Lizard	*Kentropyx striata*	GAM	3.7	-66.4	143.6	154.8	32.2	56.3	2.67		<0.001
o	Fish	*Lates calcarifer*	GLM	20.0	-31.9	69.8	73.0	25.7	19.0		0.23	0.030
p	Fish	*Neoarius leptaspis*	GAM	3.1	-32.5	74.5	81.0	15.4	26.9	2.12		0.005
q	Fish	*Hoplias malabaricus 1*	GLM	27.0	-6.2	18.5	22.5	2.6	26.0		0.66	<0.001
r	Fish	*Arapaima 1*	GAM	3.6	-30.2	70.9	81.0	9.7	48.4	2.62		<0.001
s	Fish	*Arapaima 2*	GAM	3.0	-80.6	170.5	181.6	34.3	79.0	2.04		<0.001
t	Fish	*Osteoglossum bicirrhosum*	GAM	3.5	-15.6	41.5	49.2	5.0	29.5	2.50		<0.001
u	Fish	*Cichla sp.*	GLM	29.0	-6.4	18.7	22.9	2.7	28.0		0.11	0.069
