# Supplementary material for: Body size predicts ontogenetic nitrogen stable-isotope (δ15N) variation, but has little relationship with trophic level in ectotherm vertebrate predators
Source: Sci Rep. 2024 Jun 19;14:14102. doi: 10.1038/s41598-024-61969-5 (PMC11189434; doi:10.1038/s41598-024-61969-5)
Supplement: Supplementary file 3 — Supplementary Table S2. [file 41598_2024_61969_MOESM3_ESM.pdf]

Supplementary Table S2

Body size predicts ontogenetic nitrogen stable-isotope ($\delta^{15}N$) variation, but has little relationship with trophic level in ectotherm vertebrate predators

Scientific Reports

Francisco Villamarín[1,2]; Timothy D. Jardine; Stuart E. Bunn; Adriana Malvasio, Carlos Ignacio Piña; Cristina Mariana Jacobi; Diogo Araújo; Elizângela Silva de Brito, Felipe de Moraes Carvalho; Igor David da Costa; Luciano Martins Verdade; Neliton Lara; Plínio Barbosa de Camargo; Priscila Saikoski Miorando; Thiago Costa Gonçalves Portelinha; Thiago Simon Marques and William E. Magnusson

[1]Universidad Regional Amazónica Ikiam. Grupo de Biogeografía y Ecología Espacial (BioGeoE[2]), Tena, Ecuador

[2]fco.villamarin@gmail.com

Supplementary Table S2. Summary statistics of GLM models evaluating how stomach-content-derived trophic position (TP_{diet}) vary as a function of log-transformed body mass in each predator species.

Panel in figure 2	Group	Species	Model	df.null	logLik	AIC	BIC	deviance	df.residual	Pseudo r^2	p-value
a	Crocodilian	*Caiman crocodilus*	GLM	33	-7.71	21.41	25.99	3.13	32	0.02	0.392
b	Crocodilian	*Melanosuchus niger*	GLM	20	-14.08	34.15	37.29	4.70	19	0.01	0.752
c	Turtle	*Podocnemis unifilis*	GLM	19	3.61	-1.22	1.76	0.82	18	0.03	0.448
d	Turtle	*Mesoclemmys vanderhaegei*	GLM	71	-79.53	165.06	171.89	38.40	70	0.00	0.931
e	Lizard	*Ameiva ameiva*	GLM	4	11.24	-16.49	-17.66	0.00	3	0.92	0.010
f	Lizard	*Cnemidophorus lemniscatus*	GLM	2	3.08	-0.16	-2.87	0.02	1	0.24	0.677
g	Lizard	*Kentropyx striata*	GLM	3	5.29	-4.57	-6.41	0.02	2	0.64	0.199
h	Fish	*Arapaima 1*	GLM	43	-47.03	100.05	105.40	21.84	42	0.42	0.670
i	Fish	*Hoplias malabaricus*	GLM	4	2.59	0.82	-0.35	0.10	3	0.70	0.077
