# Supplementary material for: Body size predicts ontogenetic nitrogen stable-isotope (δ15N) variation, but has little relationship with trophic level in ectotherm vertebrate predators
Source: Sci Rep. 2024 Jun 19;14:14102. doi: 10.1038/s41598-024-61969-5 (PMC11189434; doi:10.1038/s41598-024-61969-5)
Supplement: Supplementary file 4 — Supplementary Table S3. [file 41598_2024_61969_MOESM4_ESM.pdf]

Supplementary Table S3

Body size predicts ontogenetic nitrogen stable-isotope ($\delta^{15}N$) variation, but has little relationship with trophic level in ectotherm vertebrate predators

Scientific Reports

Francisco Villamarín[1,2]; Timothy D. Jardine; Stuart E. Bunn; Adriana Malvasio, Carlos Ignacio Piña; Cristina Mariana Jacobi; Diogo Araújo; Elizângela Silva de Brito, Felipe de Moraes Carvalho; Igor David da Costa; Luciano Martins Verdade; Neliton Lara; Plínio Barbosa de Camargo; Priscila Saikoski Miorando; Thiago Costa Gonçalves Portelinha; Thiago Simon Marques and William E. Magnusson

[1]Universidad Regional Amazónica Ikiam. Grupo de Biogeografía y Ecología Espacial (BioGeoE[2]), Tena, Ecuador

[2]fco.villamarin@gmail.com

Supplementary Table S3. Summary statistics of GLM models evaluating how $\delta^{15}N$ varies as a function of stomach-content-derived trophic position (TP_{diet}) in each predator species.

Panel in figure 3	Group	Species	Model	df.null	logLik	AIC	BIC	deviance	df.residual	Pseudo r^2	p-value
a	Crocodilian	*Caiman crocodilus*	GLM	26	-24.85	55.70	59.59	9.96	25	0.04	0.339
b	Crocodilian	*Melanosuchus niger*	GLM	12	-8.55	23.11	24.80	2.84	11	0.15	0.189
c	Lizard	*Ameiva ameiva*	GLM	3	3.60	-1.19	-3.03	0.04	2	0.95	0.026
d	Lizard	*Kentropix striata*	GLM	3	-4.39	14.79	12.94	2.11	2	0.08	0.718
e	Fish	*Arapaima*	GLM	48	-56.78	119.57	125.24	29.13	47	0.01	0.446
