# Supplementary material for: Body size predicts ontogenetic nitrogen stable-isotope (δ15N) variation, but has little relationship with trophic level in ectotherm vertebrate predators
Source: Sci Rep. 2024 Jun 19;14:14102. doi: 10.1038/s41598-024-61969-5 (PMC11189434; doi:10.1038/s41598-024-61969-5)
Supplement: Supplementary file 5 — Supplementary Table S4. [file 41598_2024_61969_MOESM5_ESM.pdf]

Supplementary Table S4.

Body size predicts ontogenetic nitrogen stable-isotope ($\delta^{15}N$) variation, but has little relationship with trophic level in ectotherm vertebrate predators

Scientific Reports

Francisco Villamarín[1,2]; Timothy D. Jardine; Stuart E. Bunn; Adriana Malvasio, Carlos Ignacio Piña; Cristina Mariana Jacobi; Diogo Araújo; Elizângela Silva de Brito, Felipe de Moraes Carvalho; Igor David da Costa; Luciano Martins Verdade; Neliton Lara; Plínio Barbosa de Camargo; Priscila Saikoski Miorando; Thiago Costa Gonçalves Portelinha; Thiago Simon Marques and William E. Magnusson

[1]Universidad Regional Amazónica Ikiam. Grupo de Biogeografía y Ecología Espacial (BioGeoE[2]), Tena, Ecuador

[2]fco.villamarin@gmail.com

Supplementary Table S4. Datasets of organisms used for the analyses.

Taxonomic group	Species	Locality	Country	Dataset source	Sample size	Type of data	Capture method	Obtention of stomach-contents methods	Tissue used for SIA
Crocodylia	*Crocodylus porosus*	Kakadu National Park, Northern Territory	Australia	Unpublished data	40	SIA	Baited traps	NA	Muscle, osteoderm, tail scale
Crocodylia	*Paleosuchus palpebrosus*	BR-319 Road and Piagaçu Purús Sustainable Development reserve, Amazonas	Brazil	Villamarín et al. (2018)	37	SIA	Steel snares at night from motor boat	NA	Muscle
Crocodylia	*Paleosuchus trigonatus*	BR-319 Road, Amazonas	Brazil	Villamarín et al. (2018)	45	SIA	Fyke nets set for 96 hours with rotten chicken as bait. Daily checks.	NA	Muscle
Crocodylia	*Melanosuchus niger*	Rio Javaes, Ilha do Bananal, Tocantins	Brazil	Unpublished data	18	Diet	Steel snares at night from motor boat	Stomach flushing (Legler, 1977)	Scute
Crocodylia	*Caiman crocodilus*	Purus River, Piagaçu Purús Sustainable Development Reserve, Amazonas	Brazil	Villamarín et al. (2018)	31	SIA	Steel snares at night from motor boat	NA	Muscle
Crocodylia	*Caiman crocodilus*	Rio Javaes, Tocantins	Brazil	Unpublished data	34	SIA & Diet	Steel snares at night from motor boat	Stomach flushing (Legler, 1977)	Scute
Crocodylia	*Caiman latirostris 1*	Taim Station Ecology, Rio Grande do Sul	Brazil	Unpublished data	20	SIA	Steel snares at night from motor boat	NA	Muscle

Crocodylia	*Caiman latirostris* 2	Angatuba, São Paulo	Brazil	Marques et al. (2013); Diniz-Reis et al. (2022)	31	SIA	Steel snares at night, baited traps (three iron hoops, one metre in diameter, with 6 cm mesh size) placed within the water bodies near the edge	NA	Claw
Crocodylia	*Caiman latirostris* 3	Santa Fé	Argentina	Unpublished data	76	SIA	Steel snares at night, hand for hatchlings	NA	Claw
Testudines	*Podocnemis unifilis* 1	Iriri River, Terra do Meio Ecological Station, Pará	Brazil	Unpublished data	41	SIA & Diet	Hand net (locally called landuá or puçá), and diving	Stomachs collected	Skin
Testudines	*Podocnemis unifilis* 2	Lower Amazon River, Pará	Brazil	Unpublished data	63	SIA	Trawl net (adapted at the side of the boat)	NA	Skin
Testudines	*Mesoclemmys vanderhaegei* 1	Angatuba, São Paulo	Brazil	Unpublished data	22	SIA	Steel snares at night, baited traps (three iron hoops, one metre in diameter, with 6 cm mesh size) placed within the water bodies near the edge	NA	Claw
Testudines	*Mesoclemmys vanderhaegei* 2	Chapada dos Guimaraes, Mato Grosso	Brazil	Brito et al. (2016)	79	Diet	Funnel baited traps or hand capture	(Legler, 1977)	NA
Lizard	*Ameiva ameiva*	Alter do Chão, Pará	Brazil	Magnusson et al. (1985); Magnusson (1987); Magnusson (1993); Magnusson and da Silva (1993); and Unpublished data	10	SIA & Diet	Hand	Stomachs collected	

Lizard	*Anolis auratus*	Alter do Chão, Pará	Brazil	Magnusson et al. (1985); Magnusson (1987); Magnusson (1993); Magnusson and da Silva (1993); and Unpublished data	83	SIA & Diet	Hand	Stomachs collected
Lizard	*Cnemidophorus lemniscatus*	Alter do Chão, Pará	Brazil	Magnusson et al. (1985); Magnusson (1987); Magnusson (1993); Magnusson and da Silva (1993); and Unpublished data	95	SIA & Diet	Hand	Stomachs collected
Lizard	*Kentropyx striata*	Alter do Chão, Pará	Brazil	Magnusson et al. (1985); Magnusson (1987); Magnusson (1993); Magnusson and da Silva	60	SIA & Diet	Hand	Stomachs collected

				(1993); and Unpublished data					
Fish	*Lates calcarifer*	Kakadu National Park, Northern Territory	Australia	Unpublished data	21	SIA	Gill nets set for 4-6 hours	NA	Muscle
Fish	*Neoarius leptaspis*	Kakadu National Park, Northern Territory	Australia	Unpublished data	30	SIA	Gill nets set for 4-6 hours	NA	Muscle
Fish	*Osteoglossum bicirrhosum*	Middle Juruá River, Amazonas	Brazil	Unpublished data	33	SIA	Gill nets set for 4-6 hours	NA	Muscle
Fish	*Cichla sp*	Middle Juruá River, Amazonas	Brazil	Unpublished data	30	SIA	Gill nets set for 4-6 hours	NA	Muscle
Fish	*Hoplias malabaricus* 1	Machado River, Rondônia	Brazil	Unpublished data	5	Diet	Gill nets	Stomachs collected	NA
Fish	*Hoplias malabaricus* 2	Angatuba, São Paulo	Brazil	Unpublished data	28	SIA	Gill nets	NA	Muscle
Fish	*Arapaima* 1	Purus River, Piagaçu Purús Sustainable Development Reserve, Amazonas	Brazil	Carvalho et al. (2017)	52	SIA & Diet	Gill nets	Stomachs collected	Muscle
Fish	*Arapaima* 2	Middle Juruá River, Amazonas	Brazil	Jacobi et al. (2020)	82	SIA & Diet	Gill nets set overnight and harpoon	Stomachs collected	Muscle

References

Brito, E., Souza, F., Strüssmann, C., 2016. Feeding habits of Mesoclemmys vanderhaegei (Testudines: Chelidae). Acta Herpetologica 11, 1–13.

Carvalho, F., Power, M., Forsberg, B.R., Castello, L., Martins, E.G., Freitas, C.E., 2017. Trophic Ecology of Arapaima sp. in a ria lake—river-floodplain transition zone of the Amazon. Ecology of Freshwater Fish 27, 237–246.

Diniz-Reis, T.R., Augusto, F.G., Abdalla Filho, A.L., Araujo, M.G. da S., Chaves, S.S.F., Almeida, R.F., Perez, E.B., Simon, C. da P., de Souza, J.L., da Costa, C.F.G., others, 2022. SIA-BRA: A database of animal stable carbon and nitrogen isotope ratios of Brazil. Global Ecology and Biogeography 31, 611–620.

Jacobi, C.M., Villamarín, F., Jardine, T.D., Magnusson, W.E., 2020. Uncertainties associated with trophic discrimination factor and body size complicate calculation of δ15N-derived trophic positions in Arapaima sp. Ecology of Freshwater Fish 29, 779–789.

Legler, J.M., 1977. Stomach flushing: a technique for chelonian dietary studies. Herpetologica 281–284.

Magnusson, W.E., 1987. Reproductive cycles of teiid lizards in Amazonian savanna. Journal of Herpetology 307–316.

Magnusson, W.E., 1993. Body temperatures of field-active Amazonian savanna lizards. Journal of Herpetology 53–58.

Magnusson, W.E., da Silva, E.V., 1993. Relative effects of size, season and species on the diets of some Amazonian savanna lizards. Journal of Herpetology 380–385.

Magnusson, W.E., de Paiva, L.J., da Rocha, R.M., Franke, C.R., Kasper, L.A., Lima, A.P., 1985. The correlates of foraging mode in a community of Brazilian lizards. Herpetologica 324–332.

Marques, T.S., Lara, N.R., Bassetti, L.A., Piña, C.I., Camargo, P.B., Verdade, L.M., 2013. Intraspecific isotopic niche variation in broad-snouted caiman (Caiman latirostris). Isotopes in Environmental and Health Studies 49, 325–335.

Villamarín, F., Jardine, T.D., Bunn, S.E., Marioni, B., Magnusson, W.E., 2018. Body size is more important than diet in determining stable-isotope estimates of trophic position in crocodilians. Scientific Reports 8, 2020.
